# Œdème papillaire de stase bilatéral révélant une hypertension intracrânienne bénigne

**DOI:** 10.11604/pamj.2014.18.96.4229

**Published:** 2014-05-27

**Authors:** Hakima Elouarradi, Rajae Daoudi

**Affiliations:** 1 Université Mohammed V Souissi, Service d'Ophtalmologie A de l'hôpital des spécialités, Centre hospitalier universitaire, Rabat, Maroc

**Keywords:** Œdème papillaire, hypertension intracrânienne, cécité, papilledema, intracranial hypertension, blindness

## Image en medicine

L'oedème papillaire est l'expression clinique d'affections diverses. Il s'agit d'un gonflement liquidien et / ou axonal de la tête du nerf optique du à un blocage du flux axoplasmique au niveau de la lame criblée. Il s'agit d'une patiente âgée de 37 ans, obèse, sous contraception orale depuis 3 ans, qui s'est présentée aux urgences neurologiques avec des céphalées, des acouphènes, et des éclipses visuelles. L'examen neurologique est normal. L'examen ophtalmologique a objectivé une acuité visuelle corrigée de loin à 9/10 au niveau des 2 yeux, l'examen du segment antérieur est normal en ODG avec au fond d'oeil un oedème papillaire bilatéral de stase. Patiente ayant bénéficié d'une angio IRM qui est revenue normale. La ponction lombaire réalisée en décubitus latéral objectivant un LCR de composition normale avec pression élevée de 27 cm H2O. Le diagnostic d'hypertension intracrânienne bénigne est posé. La contraception orale est arrêtée et la patiente adressée pour consultation endocrinologie pour rechercher d'autres facteurs de risque et pour un éventuel rééquilibrage alimentaire pour perte de poids. Patiente ayant bénéficié de ponctions lombaires à but thérapeutique, mise sous acétazolamide par voie orale avec une nette amélioration de ses acouphènes. L'hypertension intracrânienne bénigne est une pathologie fréquente, mais qui reste un diagnostic d’élimination, terrain de prédilection la femme jeune avec excès de poids sous contraception orale, se présentant dans un tableau d'hypertension intracrânienne, une acuité visuelle généralement longtemps conservée avec un oedème papillaire bilatéral de stase pouvant conduire à la cécité par atrophie optique si retard diagnostic et prise en charge.

**Figure 1:**
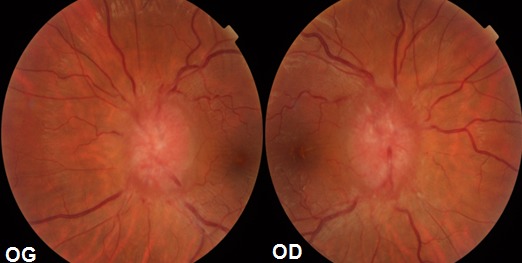
Œdème papillaire de stase bilatéral

